# A Mobile Intervention to Improve Uptake of Pre-Exposure Prophylaxis for Southern Black Men Who Have Sex With Men: Protocol for Intervention Development and Pilot Randomized Controlled Trial

**DOI:** 10.2196/15781

**Published:** 2020-02-20

**Authors:** Anne-Emilie Rouffiac, Laura Whiteley, Larry Brown, Leandro Mena, Lacey Craker, Meredith Healy, Kayla Haubrick

**Affiliations:** 1 Young Adult Behavioral Health Program Rhode Island Hospital Providence, RI United States; 2 Department of Psychiatry and Human Behavior Warren Alpert Medical School of Brown University Providence, RI United States; 3 Department of Population Health Science University of Mississippi Medical Center Jackson, MS United States; 4 Division of Infectious Diseases Department of Medicine University of Mississippi Medical Center Jackson, MS United States

**Keywords:** pre-exposure prophylaxis (PrEP), HIV prevention, men who have sex with men (MSM)

## Abstract

**Background:**

The uptake of pre-exposure prophylaxis (PrEP) has been slow for young black men who have sex with men (BMSM) living in the southern United States. This is a significant issue because 8 of the 10 states with the highest rates of new HIV infections are in the South. Jackson, Mississippi (MS), the site of this project, has the second highest AIDS diagnosis rate in the nation and the highest rate of HIV infection for young, urban BMSM. This study will develop and test an engaging, interactive, and cost-effective mobile messaging intervention to improve engagement in PrEP care for BMSM aged 18 to 35 years living in Jackson, MS.

**Objective:**

The goals of this mixed methods study are to (1) conduct qualitative interviews with young BMSM in Jackson, MS, to understand individual, community, and structural barriers affecting engagement in PrEP-related care, (2) assemble a PrEP mobile messaging intervention that includes text messages with publicly available internet content (websites and YouTube videos) that provide factual information, motivational materials, and behavioral skills related to PrEP and HIV prevention, and (3) evaluate the preliminary efficacy of the intervention in a randomized controlled study with PrEP-eligible BMSM receiving care in STI/HIV testing clinics in Jackson, MS.

**Methods:**

This research protocol will be conducted in 2 phases. A development phase will involve in-depth interviews (n=30) with PrEP-eligible BMSM who receive care in STI/HIV testing clinics in MS. These interviews will allow researchers to select the texted material that will be sent out during the intervention. The second phase will consist of an unblinded, small, randomized controlled trial among 66 new participants to examine the preliminary efficacy of the intervention compared with enhanced standard of care (ESC) on attendance at a PrEP services appointment (the first step in initiating PrEP care) and receipt of a PrEP prescription, based on self-report and electronic medical records. The free, publicly available material will be sent to PrEP-eligible BMSM in 8 to 16 interactive text messages over 4 weeks. Study assessments will occur at enrollment and at 4- and 16-weeks postenrollment and can be completed online or in person. All participants will be recruited from a local clinic.

**Results:**

Institutional review board approval was received on January 16, 2017, and research activities, subsequently, began in February 2018. Recruitment for the study concluded in November 2019. In total, 65 participants were randomized with 33 being assigned to the intervention and 32 to ESC. Collection of follow-up data is ongoing.

**Conclusions:**

This PrEP mobile messaging intervention aims to increase uptake of PrEP by BMSM in the southern United States. This intervention uses interactive, tailored text messaging and appealing free Web content (publicly accessible educational websites and YouTube videos) to promote linkage to PrEP care and increase HIV preventative behaviors. A cost-effective PrEP mobile messaging intervention has great potential to improve information about PrEP, improve motivation to use PrEP, and decrease stigma and structural barriers that often prevent engagement in PrEP-related medical care.

**Trial Registration:**

ClinicalTrials.gov NCT03308097; https://clinicaltrials.gov/ct2/show/NCT03308097

**International Registered Report Identifier (IRRID):**

DERR1-10.2196/15781

## Introduction

### Background

The strategy of using antiretrovirals as a form of HIV prevention, known as pre-exposure prophylaxis (PrEP), has received considerable attention and holds tremendous promise [1,2]. Despite the advancements supporting PrEP efficacy and its availability, uptake of PrEP by many men who have sex with men (MSM) has been slow. In particular, uptake has been slow for young black MSM (BMSM) who live in the southern United States [3-8]. This is a significant issue because BMSM living in the South have particularly high rates of HIV. Eight of the ten states with the highest rates of new HIV infections are located in the South, and estimates suggest that BMSM in the South are 5 times more likely than white MSM in the South to become infected with HIV [9]. The site of this proposed project, Jackson, MS, has the highest prevalence of HIV among urban MSM living in the United States (39.5 per 100 MSM) [9]. The premise of this project is to develop a targeted mobile messaging intervention to improve linkage to PrEP care for young BMSM in Jackson, MS, who are at high risk for HIV infection. Interventions that improve linkage to PrEP care are urgently needed for BMSM in the South.

Truvada (tenofovir disoproxil fumarate/emtricitabine) for PrEP was approved by the US Food and Drug Administration as an HIV prevention method in July 2012 for individuals aged 18 years and older and in 2018 for adolescents weighing at least 77 pounds [10]. Despite this, knowledge and access to information about PrEP remains low in many MSM communities. This has restricted linkage and uptake of PrEP and, ultimately, the effectiveness of PrEP at the community level [3-7,11-13]. To use PrEP, individuals must have accurate knowledge, understand its risks and benefits, and be willing to take it. Studies show that many high-risk individuals who would be excellent candidates for PrEP have not taken it simply because their knowledge about it is limited [3,11]. In one study conducted among young MSM in 2013, only 27% of study participants were aware of PrEP [11]. Knowledge about PrEP has increased recently [13,14]; however, some studies show that awareness remains markedly lower among men of color, those who live in southern and rural areas in the United States, and those whose primary care providers are not aware that they have sex with men [15]. Among 436 BMSM surveyed in Atlanta, GA, from January 2012 (6 months prior to PrEP approval) to March 2014 (20 months after approval), only 20.5% were aware of PrEP before approval and only 23.4% were aware of PrEP after approval [3]. Improving southern, young BMSM’s knowledge about PrEP is critical for successful linkage to PrEP care.

Strengthening the relationships young BMSM have with providers is critical for successful PrEP uptake. Young BMSM who are at highest risk of HIV infection are historically underserved by the health care system [16-19]. Therefore, engaging patients in care is challenging and requires support for doctors and patients [19,20]. Brooks et al [6] found that heightened concerns over potential side effects to PrEP pose a significant barrier to engagement and linkage to PrEP care for BMSM. In the southern United States, homophobia, stigma, and clinician/patient communication all influence initiation of PrEP [21-23]. Eaton et al [3] surveyed 398 HIV negative BMSM at a black Gay Pride event in the southeastern United States. Among this sample, 60% agreed that they were uncomfortable talking to a health care provider about having sex with men. Race-based medical mistrust was also identified as a barrier to engaging in PrEP. Around 1 in 5 participants reported that “people of my race cannot trust doctors and health care workers” (21%), “people of my race should be suspicious of information from doctors and health care workers” (19%), and “people of my race should be suspicious of medicine” (19%). Strengthening communication and connection between medical caregivers and the young BMSM community are crucial components to improving linkage and initiation of PrEP care in the South [12,14,18,23]. Accurate information about PrEP and issues such as potential side effects will need to be shared in a method that considers a history of mistrust to effectively allay concerns among southern BMSM [18].

Furthermore, addressing structural/economic barriers to receiving PrEP-related care is imperative for BMSM. Effective interventions to improve PrEP must assist individuals when navigating insurance companies and enrolling in medication co-pay assistance. In 2014, approximately 21% of African Americans did not have health insurance, compared with 12% of whites [24]. The situation is particularly concerning for southern BMSM since some of the highest levels of poverty in the United States are in the South. Mississippi has the highest poverty level (28%) in the United States, which directly impacts health care access. Southern states also have the most restrictive Medicaid eligibility criteria and provide fewer Medicaid benefits than other regions in the country [25]. Interventions that help navigate the economics of getting on PrEP, such as PrEP co-pay and full medication assistance programs, are essential to the successful implementation of PrEP in the South, where poverty is highest.

Last, decreasing stigma about PrEP and HIV can improve linkage and initiation of PrEP-related care for BMSM. Participants in PrEP demonstration studies have reported that stigma affects their decision to initiate PrEP care [19,20,26-30]. Gay men have reported a fear of being labeled as promiscuous by doctors and “Truvada whores” by friends. These stigmatizing labels infer that PrEP is associated with unbridled sex and may be a barrier to care [20,27,30]. Furthermore, the only currently available PrEP method, Truvada, is the same medication used to treat those infected with HIV. In the South, where HIV-associated stigma is high and HIV is prevalent, being seen with Truvada can be perceived as being HIV-infected, which is a further barrier [19,30]. HIV-related stigma is particularly widespread in the South [11,12,20,31]. Interventions must replace negative community attitudes about individuals using PrEP with positive impressions of PrEP users as individuals who take care of themselves and others [32].

PrEP and HIV prevention interventions for young BMSM need to be interactive, cost effective, and easily integrated into existing clinical care. We will use interactive, tailored text messaging and appealing free Web content (publicly accessible educational websites and YouTube videos) to promote linkage to PrEP care and increase HIV preventative behaviors. A cost-effective, interactive intervention that uses mobile technology is particularly compelling for use with young MSM. MSM make greater use of cell phone technologies than heterosexuals [33], and there is evidence that mobile phone teledensity (ie, number of phones per person) in black southern communities has outpaced black communities in the northeast [34-37]. On a monthly basis, young black adults in the United States spend nearly 56 hours using smartphone apps or internet browsers and 2.5 hours watching videos on their smartphones [38]. With such widespread use of mobile technologies, it is not surprising that data supports the use of mobile technology to improve health [39-41]. Meta-analyses have shown that mobile interventions are most effective when they are interactive and tailored [39-41]. The widespread appeal and use of smartphones and promising data supporting mobile messaging interventions for health promotion create a unique opportunity. Information about PrEP and HIV prevention can be delivered to young, southern BMSM during their leisure time, outside of the sexually transmitted infection (STI)/HIV clinic, and in a manner that is cost effective and easily scalable [34,35,38,42-46].

### Theoretical Framework for Intervention

The information-motivation-behavioral skills (IMB) model is a well-established conceptualization for improving engagement in care and decreasing HIV risk behaviors. HIV prevention interventions and linkage/engagement in care interventions based on the IMB model have demonstrated efficacy [47,48]. Reviews have suggested that interventions guided by theory are more efficacious than those not driven by theory [47-50]. According to the IMB model, health information, motivation, and behavioral skills are the fundamental determinants of health behavior. For a PrEP-related intervention to be successful, a person must learn information that is directly relevant to HIV prevention and treatment with PrEP. Knowledge is a necessary but not sufficient condition for change. Personal motivation to engage in HIV preventative behavior or engage in treatment regimens (attitudes about health) and motivation (perceived social, cultural, and structural support for performing these acts) are essential for change. Finally, skills for performing healthy behaviors and a sense of self-efficacy must be easily applied to an individual’s cultural, social, and structural setting [47-50]. Engagement in PrEP care can be facilitated by accurate knowledge of medication benefits, self-efficacy for care, and structural support [51,52]. Our intervention, and the online material it is composed of, will address these factors within the IMB model and the local/geographic, structural, and racial context. The IMB model is broadly applicable and can be used to organize and guide theoretically consistent and culturally and structurally informed intervention content [53].

### Aims and Objectives

This mixed methods study will develop an online PrEP mobile messaging intervention for PrEP-eligible BMSM at the STI/HIV testing clinics in Jackson, MS. The intervention will be created with the help of in-depth interviews with 30 young BMSM in MS that will seek to understand individual, community, and structural barriers affecting IMB factors relevant to engagement in PrEP-related care. We will then conduct a randomized controlled pilot study with 66 PrEP-eligible BMSM seen at STI/HIV testing clinics. This pilot will evaluate the preliminary efficacy of the PrEP mobile messaging intervention compared with enhanced standard of care (ESC) on improving HIV-related knowledge and attitudes, attendance at a PrEP services appointment, and receipt of a PrEP prescription. These data will provide preliminary evidence of the intervention’s impact and inform a larger randomized controlled trial (RCT), if needed, as a test of the intervention.

## Methods

### Trial Registration and Institutional Review Board Approval

The research and ethics presented in this study were approved by the institutional review board of Rhode Island Hospital and University of Mississippi Medical Center (UMMC). This study is registered on ClinicalTrials.gov (NCT03308097).

### Design

This research protocol will be conducted in 2 phases. A development phase will involve in-depth interviews (n=30) with PrEP-eligible BMSM who receive care in STI/HIV testing clinics in MS. Interviews will be conducted by LB, LW, or a trained senior research associate in a private area of the UMMC STI/HIV testing clinic. These interviews will allow researchers to select and adapt the material that will be sent out through the PrEP mobile messaging intervention. The material will be composed of free, publicly available links to websites and YouTube videos that provide factual information, motivational materials, and behavioral skills related to PrEP and HIV prevention. The second phase will consist of a small, unblinded, parallel, randomized controlled pilot study among 66 new participants in the clinics to examine the preliminary efficacy of the intervention compared with ESC on attendance at a PrEP services appointment and receipt of a PrEP prescription. Participants will be randomized using the block randomization method designed to randomize subjects into groups that result in equal sample sizes (1:1). This method is used to ensure a balance in sample size across groups over time. Blocks will be small and balanced with 6 participants assigned to each block. Blocks will be generated using an internet application and saved on a secure drive accessible only to Rhode Island Hospital staff. A research assistant at Rhode Island Hospital will randomize participants after they are enrolled by UMMC staff. The intervention material will be sent to PrEP-eligible BMSM in 8 to 16 interactive text messages over 4 weeks. Study assessments will occur at enrollment and at 4 and 16 weeks postenrollment.

### Participants

Young BMSM, aged 18 to 35 years, who visit the STI/HIV testing clinics in Jackson, MS, and who are eligible for PrEP according to current treatment guidelines [54] will be eligible for enrollment in each phase of the study according to the following criteria: (1) English speaking, (2) eligible to receive prophylactic antiretroviral treatment, (3) not enrolled in another PrEP-related study or HIV prevention study, and (4) able to give consent/assent and not impaired by cognitive or medical limitations as per clinical assessment. Clinical assessment will occur by members of the proposed research team, as they have substantial prior clinical (medical and psychiatric) and research experience in care of young adults and adults. While having a smartphone or computer is not an eligibility criterion, participants without regular access to this technology must be willing to come to the clinic twice a week if they are randomized to the intervention arm to access intervention content. There will not be overlap between subjects in the developmental and RCT phases. We are enrolling only BMSM between the ages of 18 to 35 years, because they are the subgroup most at risk for acquiring HIV in Jackson, MS. Limiting the study to young BMSM will allow for the development of a mobile intervention that is targeted, acceptable, and engaging for this specific population.

### Description of Intervention Content

The PrEP mobile messaging intervention employs graphics, characters, and video content specifically chosen to be appealing to young BMSM. Text messages and accompanying Web content will target IMB constructs. These IMB-consistent messages and content all aim to increase knowledge and engagement in PrEP care and decrease HIV risk behaviors. Examples of the IMB-informed texts include: “Do you want to see a cool website with more information about PrEP? Check out this website” which provides information, “Hear from Dr. Mena, who works in Jackson. You can take PrEP and still have fun” which enhances motivation, and “Hear tips for taking PrEP each day and how to get PrEP easily in Jackson” which teaches behavioral skills.

**Figure 1:**
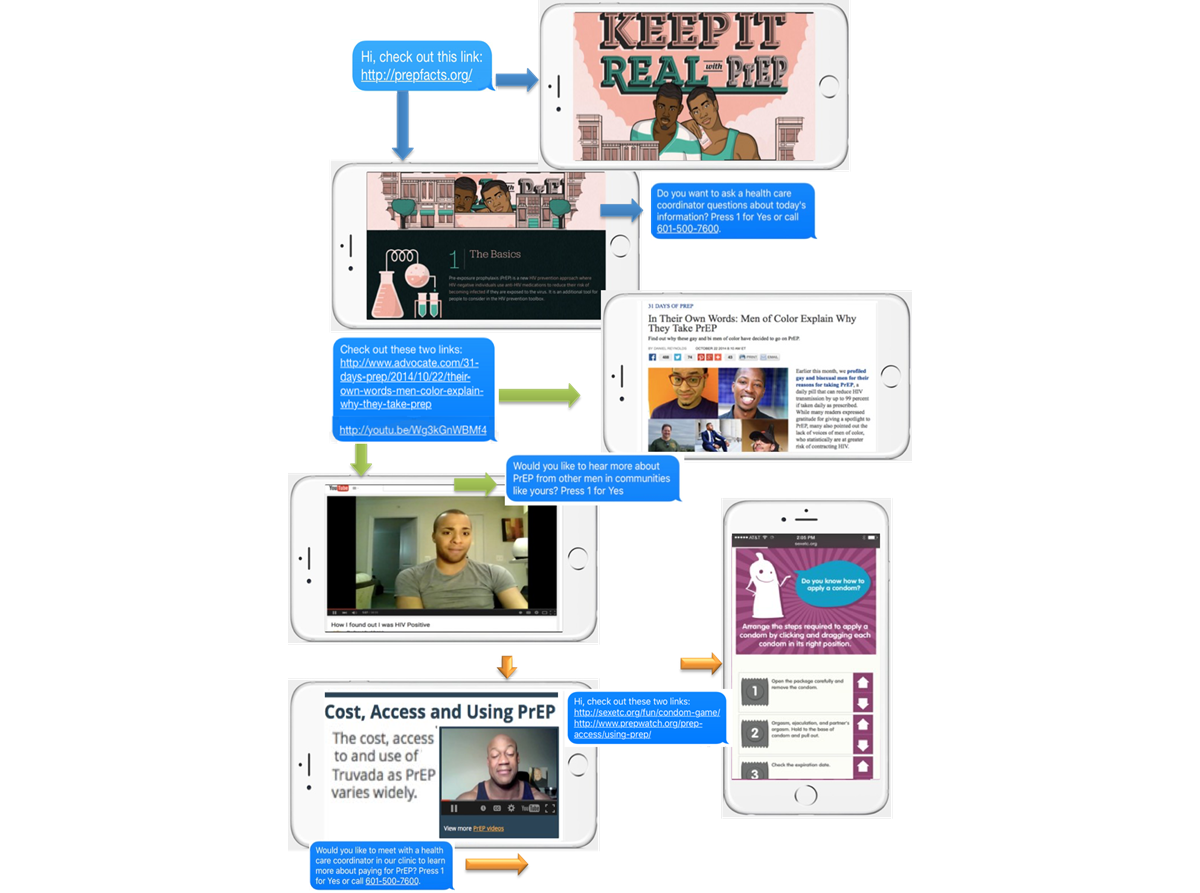
Preliminary intervention text messages developed from publicly available internet content.

Interviews will ensure that the intervention material is relevant to community context (eg, stigma, structural barriers). Currently, the intervention is composed of 8 text messages with 1 to 2 links to publicly available Web content in each message. Participants will be sent 2 text messages per week over 4 weeks. The timing and length of the texts and Web activities are based on the timing and length of the efficacious Center for AIDS Research HIV prevention project reviewed in preliminary studies [55]; however, the length and frequency of the intervention and intervention activities will be assessed and edited during the development stage. Each text message will have a link to Web information, quizzes, and games focused on providing facts about PrEP and HIV transmission and prevention and on correcting misperceptions about HIV and PrEP. Videos that are publicly available online and that specifically address engagement in PrEP-related care and HIV prevention information deficits identified with BMSM will be part of the modules. Pictures and graphics have been selected to appeal to BMSM. Young, black male role models are used to impart information and address attitudes, stigma, and social norms concerning engagement with providers, PrEP, and HIV risk. Accurate information will reduce PrEP misconceptions that fuel stigma. Videos that show BMSM discussing the positive aspects of PrEP for them, their partners, and their communities will also help reduce stigma. The intervention will also address structural barriers. Mobile-ready information has been selected to help participants navigate health insurance, medication assistance programs, and co-pay assistance programs. Figure 1 shows sample text messages with pictures of accompanying IMB Web content.

Preliminary material will either be confirmed or adapted following in-depth interviews with 30 BMSM eligible for PrEP seen at STI/HIV testing clinics in Jackson, MS. Thus, these interviews finalize the links and digital material that will be sent out by the mobile messaging intervention during the RCT.

### Developmental Phase Qualitative Interviews

Qualitative interviews will be conducted by LB, LW, or a trained senior research associate, who have experience with interviews used to develop behavioral interventions and health care provider training. Interviews will be digitally recorded and recordings uploaded to a secure file location on the Rhode Island Hospital server that will only be accessible to project staff.

Developmental phase participants will be recruited from the UMMC STI/HIV testing clinics. They will be screened for eligibility by UMMC research assistants by phone or in person. Those eligible and interested will arrive the day of the focus group or interview, and research staff will review the consent form with them. Those who sign the consent form will proceed with the interview.

Interview questions were developed to reflect the IMB model. Questions focus on the relevance and utility of the presented content and its appeal and will solicit suggestions for potential improvements. Feedback will be elicited after exposure to mobile material in a secure clinical space at the UMMC STI/HIV testing clinics. We will ask about general reaction to the preliminary Web content and all texted queries. Participants will be asked about the acceptability and relevance of texts and mobile content, actions, and graphics as they relate to race, culture, and structural factors. We will also concentrate on deeper, or more complex, emerging themes. For example, if there are particular barriers to engagement in PrEP care, such as misconceptions about the perceived risk of HIV, more content will target this misperception [56-58]. If other barriers to engagement in PrEP-related care, such as misinformation about side effects or mistrust of the medical community, are identified, more content will target these issues. For example, participants will be asked “As a black, gay male, how worried are you about getting HIV? How effective do you think PrEP is at preventing someone from getting HIV?” and “How comfortable do you feel talking to your doctor about sex, HIV, and PrEP?” After viewing preliminary Web content, participants will be asked, “Which parts of this website or YouTube video would influence how concerned you are about HIV?” “Do any parts of this website or video help you feel prepared to start PrEP?” “Do any parts of this website help you to know how to pay for PrEP?” and “How could these messages be made more relevant to you, your friends, and your partners?” Major topics and subtopics from the interviews will be coded. Additional codes will be generated for topics that invariably arise and that may have significance to the project. Qualitative data will be analyzed using the qualitative software package NVivo version 11 (QSR International). In order to maintain scientific adequacy and confirmability, we will perform semistructured interviews on a sufficient number of participants from each relevant subgroup defined by age (divided at 25 years); level of interest in starting PrEP; and identifying as gay, bisexual, or neither until there is redundancy in themes and general feedback. All interviews will focus on how sexual orientation, race, stigma, culture, age, and structural factors influence IMB for the major targets of the intervention.

After the first 15 qualitative interviews, another draft preliminary PrEP mobile messaging intervention will be assembled. This draft intervention will be assessed with another group of young BMSM using the similar qualitative procedures. Interviews will continue until thematic redundancy is achieved. These iterative design efforts will provide an intervention that is culturally acceptable and theoretically informed. Throughout the developmental phase, members of the research team will iteratively review and assess the clinical utility of all intervention components via the BMSM participants’ qualitative feedback and audiotaped session materials. The team will assess the strengths and weaknesses of intervention components and indicate revisions. Specifically, we will assess the intervention content’s accuracy and suitability, ability of the intervention content to engage MSM, and ability to target STI/HIV-related behavioral change with material and techniques consistent with the IMB model. Additionally, the team will review the intervention material for relevance, conceptual clarity, and potential technological problems.

### Pilot Randomized Controlled Trial

#### Overview

We will evaluate the impact of the IMB mobile PrEP/HIV intervention compared with ESC in an RCT with 66 newly recruited BMSM. Recruitment will take place at local STI/HIV testing clinics using flyers and word of mouth. Flyers list the eligibility criteria and provide contact information of the UMMC research assistant they can contact if they would like to participate in a study to test a new mobile messaging tool that provides information about PrEP. Those interested in participation will be screened either in person or over the phone by research assistants in MS. Those interested and meeting all eligibility requirements will be scheduled to complete consent and baseline assessment if it does not occur on the same day. Research staff will then go over the consent form (Multimedia Appendix 1) in a private area of the STI/HIV testing clinic, and participants will fill out the consent and locator forms. Participants will then complete the baseline survey and be placed in either the intervention or ESC group using computerized, block randomization. We will compare conditions on attendance at a PrEP services appointment and receipt of a PrEP prescription at 16 weeks using data from the electronic medical record and by self-report. Conditions will also be compared by self-report on PrEP and HIV-related knowledge, attitudes, skills, and risk behaviors at baseline, 4 weeks (immediately postintervention), and 16 weeks.

#### Pre-Exposure Prophylaxis Mobile Messaging Intervention

Participants in the intervention will receive text messages with intervention content over 4 weeks on their smartphones. Mobile phones will not be provided by the researchers; therefore, participants must have their own working smartphone or computer or be willing to come into the clinic regularly to access content to be eligible to participate. The intervention is composed of 8 to 16 texts with links to Web content. Texts will be sent twice a week over 4 weeks by a research assistant using the Health Insurance Portability and Accountability Act (HIPAA)-compliant Study Management and Retention Toolkit (SMART) developed by Emory University [59]. While there is minimal human involvement in the intervention, participants have the option to contact research staff by phone or SMART if they have any questions or concerns. Based on feedback from the developmental phase, intervention content was selected to be less tailored to the population and more diverse and inclusive. Videos were also included that were perceived as more accurate representations of social interactions in the south. The preliminary timing and length of the texts and Web activities are based on the timing and length of the efficacious Center for AIDS Research HIV prevention project reviewed in preliminary studies (PI: LW). Each mobile link sent to participants will take between 5 and 10 minutes to read, listen to, or interact with. Participants can engage with text message material on their phones at any place that is convenient and comfortable.

#### Enhanced Standard of Care Condition

**Figure 2:**
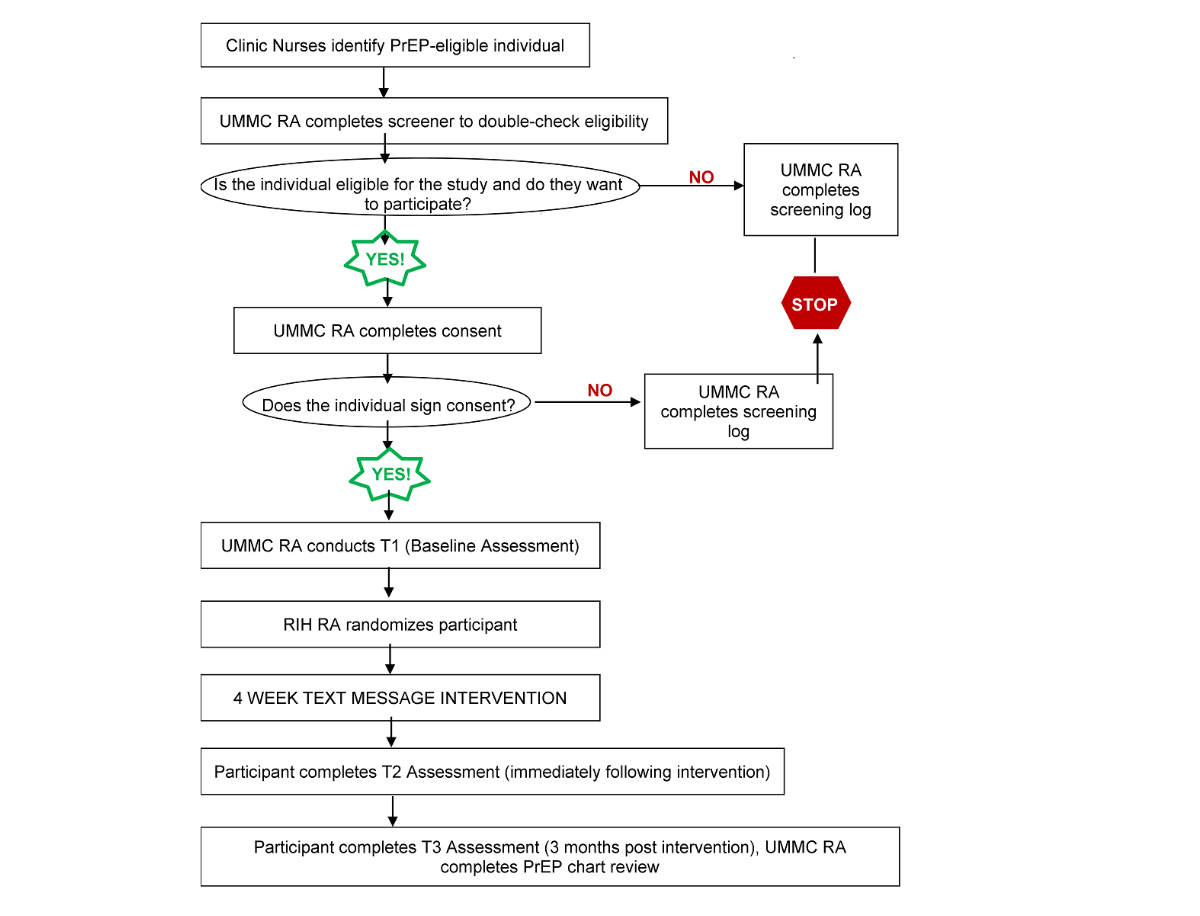
Study flow diagram of the randomized controlled trial phase.

The impact of the intervention will be compared with ESC. At both STI/HIV testing clinics in which subjects are recruited, the medical director and his staff assess patients for PrEP eligibility and HIV risk (as defined by the Centers for Disease Control and Prevention [CDC] guidelines) [54]. Feedback is given to each patient on their current risk behavior and future plans (personal risk summary). Patients are given a CDC informational handout with basic PrEP facts; shown a brief, 5-minute, free/publicly available online video about PrEP; and given contact information for the clinic care coordinator. These procedures are consistent with CDC guidelines for PrEP, and because of the handout, personal risk summary, and video, the procedure can be considered ESC. The medical director and his team have used the handout and video for the past 2 years. The video is colorful, informative, and well received but has not been chosen with specific qualitative feedback from BMSM. Patients in both conditions will receive the ESC clinical encounter described here and be followed for assessments. Only patients in the IMB mobile messaging intervention will receive the additional 8 to 16 text messages (Figure 2).

### Measures and Assessments

#### Assessments

Assessments will occur at baseline, immediately postintervention (4 weeks), and 3 months postintervention (16 weeks) and will take 45 minutes to complete.

#### Measures

A computer self-interview using Research Electronic Data Capture (REDCap) will be used to assess behavior since it is confidential, allows for complex branching/skip patterns, and detects greater rates of risk behavior [60]. Participants will be emailed the REDCap surveys 2 days prior to their appointments, but they may also opt to complete the surveys on a computer at the UMMC STI/HIV testing clinics. Standard items will be administered to gather demographic data (including age, educational level, sexual orientation, socioeconomic status, race, ethnicity, and stability of housing). The measures below will be used to evaluate HIV- and PrEP-related knowledge, attitudes, and behavior (see Table 1 for linkage between constructs and intervention).

**Table 1:** Linkage between constructs, intervention foci, and assessment instruments.

IMB^a^ construct	Description of construct	Intervention foci	Assessment instruments
Information	PrEP^b^ and HIV risk knowledge	Increased HIV/PrEP knowledge, perceived vulnerability to HIV/STIs	HIV/PrEP Knowledge Scales
Motivation	Risk attitudes, intentions, and self-efficacy for starting PrEP and engaging in PrEP-related medical care	Improved motivation for PrEP and related medical care, increased motivation for safer sex, decreased perceived structural and stigma barriers	IMB PrEP Care Motivation Scale, Self-Efficacy for PrEP Care, Rollnick’s Readiness Ruler
Behavior	Attendance at PrEP prevention appointment, sexual risk	PrEP prevention appointment, safer sex skills	Attendance at PrEP services appointment, receipt of PrEP prescription, and sexual risk self-report

^a^IMB: information-motivation-behavioral skills.

^b^PrEP: pre-exposure prophylaxis.

### Outcomes

#### Primary Outcome

With participant consent, staff at the STI/HIV testing clinic will abstract from the electronic medical record any PrEP services appointment and any PrEP prescription received around 20 weeks (4 weeks after final assessment). Participants will also be asked to self-report if they are currently taking PrEP for each assessment. This study is occurring in the only two PrEP clinics in Jackson, and both are affiliated with UMMC. Participants are recruited from the STI/HIV clinics run by the same medical staff. PrEP is unavailable elsewhere in Mississippi, so virtually no one will begin PrEP at another location. While study inclusion criteria do not restrict to only those living in Jackson, MS, we anticipate that accessibility to the recruitment sites will lead to a predominately local sample.

#### Secondary Outcomes

The following self-report scales on secondary outcomes will be asked on all three questionnaires. We will assess how responses to these measures change over time.

#### HIV/Sexually Transmitted Infection and Pre-Exposure Prophylaxis Knowledge

The HIV Knowledge Scale assesses knowledge about issues such as risks for HIV using 18 items with true, false, or do not know response options. Test-retest reliability (*r*=.73) and internal consistency (reliability coefficient=.90) were both satisfactory in studies with at-risk young adults [61]. The STI Knowledge Questionnaire uses similar response options with 10 items assessing risk for and treatment of sexually transmitted infections. Test-retest reliability (*r*=.88) and internal consistency (reliability coefficient=.86) were both satisfactory in studies with at-risk young adults [62]. Because there is no PrEP knowledge scale with published psychometrics, we will use a 15-item questionnaire to assess knowledge (true/false/don’t know) based on facts from the CDC and the San Francisco AIDS Foundation websites concerning PrEP. The items will be tested for readability and relevance with the target population in the qualitative phase of the study. Items will be revised if needed for the RCT portion of this study.

#### Personal and Social Motivational Readiness for Pre-Exposure Prophylaxis Care

Rollnick’s Readiness Ruler [63] will be used to assess motivation for engaging in PrEP care. Respondents rate how ready they are to (1) attend a PrEP services appointment, (2) begin PrEP, and (3) go to PrEP-related medical appointments on a scale from 1=not ready to 10=ready to engage. Participants will also complete the 10-item, Likert-style IMB PrEP Motivation Scale from the LifeWindows Project Team. It has been modified to assess personal and social (culture and structure) motivations for PrEP rather than antiretroviral therapy and is used in our ongoing study of PrEP adherence (1R34 MH104068) in consultation with Dr Jeffery Fisher, one of the developers of the IMB model [64].

#### Pre-Exposure Prophylaxis and Appointment Self-Efficacy

This measure was developed based on Bandura’s theory of self-efficacy [65] and was shown to have strong reliability (alpha>.80) [66]. The instrument consists of Likert-style items (with 5 response options). Three items assess self-efficacy for taking PrEP as prescribed and attending PrEP-related medical appointments. The IMB PrEP Behavioral Skills Scale has 14 Likert-style items (modified to address PrEP rather than antiretroviral therapy). This measure assesses information, motivation, and perception of behavioral ability to perform the necessary PrEP skills. It has an internal consistency of .90 when used with infected adults [64].

#### Risk Behavior Assessment

The risk behavioral assessment (used in LB’s other federally funded projects) is a reliable and valid computer-assisted structured interview assessing self-reported sexual behaviors. It assesses types of sexual behavior (ie, anal, oral, vaginal) in the past 3 months, frequency of sex, age of sexual debut, and number and gender of partners. Additional questions cover use of barrier method contraception, sex with high-risk partners, transactional sex, reasons for condom nonuse, frequency and quantity of substance use, and having sex while using alcohol and drugs [67].

### Statistical Analysis

#### Hypothesis One

The PrEP mobile messaging intervention, developed with participant feedback, will be judged by participants to be feasible, appealing, relevant, and useful. Intervention links that receive a mean score of less than 30 on the session evaluation form or less than 24 on the client satisfaction questionnaire will be discarded.

#### Hypothesis Two

A total of 66 participants will be randomized to 2 conditions. Those in the IMB PrEP mobile messaging intervention condition will show improvements in each of the IMB domains: information (HIV and PrEP knowledge), motivation (motivational readiness for PrEP and medical care, improved self-efficacy, and improved attitudes for PrEP and medical treatment), and behavior (HIV risk behavior score and engagement in PrEP). Information, motivation, and HIV risk scores will be evaluated at 4 and 16 weeks using linear mixed-effect models, which will account for the 3 assessments (0, 4, and 16 weeks) being nested within individuals [68,69]. We will test for differences in linear change over time between intervention and control groups on each outcome variable. For the PrEP uptake outcome, we will evaluate difference between conditions in the proportion of participants who engaged in PrEP (attended a PrEP medical visit, received a PrEP prescription, or self-reported starting PrEP). Propensity scores (ie, inverse probability of treatment weighting) will be used to account for any imbalance in baseline characteristics between conditions [70]. Hierarchical linear modeling analyses are robust if missing data amount is small. If missing data amount is larger, last observation carried forward techniques will be used for missing scale data. Outcomes such as engaging in PrEP care (attending a PrEP medical visit or receiving a PrEP prescription) can be obtained from the medical record regardless of subject attrition.

As this is an intervention development study and the impact of the experimental intervention is not known, there may not be adequate power to determine the efficacy of the IMB PrEP mobile messaging intervention, and pilot studies are not designed to provide accurate estimates of effect sizes upon which to base large trials [71]. Nevertheless, the small pilot RCT may provide a signal of impact on its major outcomes. We conservatively assume that retention of 66 participants over 16 weeks is 85% based on previous clinic trials. Power analyses were run with Optimal Design 3.01 (open source, University of Michigan) [72]. The model assumed 3 assessments nested within cases. The hierarchical linear modeling analyses with alpha of 0.05 and power of 0.80 will be able to detect an effect size of 0.38 SD for change in the information and motivation outcomes. If the alpha is 0.10 (as is appropriate in exploratory research), then the power is 0.80 to detect an effect size of 0.34 SD. Power to detect a difference in the proportion of participants beginning PrEP by 16 weeks is 0.80 if 10% of the ESC group begin PrEP (similar to our preliminary studies) and 39% of the intervention group begin PrEP. Because chart review will extend to 24 weeks, actual power should be somewhat greater.

### Incentives

For their involvement in this study, developmental phase participants will receive $50 gift cards as an incentive. During the RCT, subjects will receive $50 gift cards for completing the baseline assessment and $40 for the 4- and 16-week assessments. Participants who confirm contact information and research appointments prior to the 4- and 16-week assessments will receive an additional $10 per appointment. Receipts for this payment are offered to participants should they deem it necessary.

### Ethical Considerations

To protect participants from potential risk of breach in confidentiality, the following measures will be taken. Participant research data will be identified by numeric ID only and any records containing potentially identifying information will be kept separate from any research data. All research data (written records and audiotapes of program sessions) will be kept in a locked file, and electronic data will be password-protected. All of the study-related materials will only be accessible to research staff. All data collection will take place in secure and supervised clinical settings or with HIPAA-compliant software (REDCap). All study personnel have completed training and received certification in Human Subjects Research Protection (Collaborative Institutional Training Initiative Program) and HIPAA regulations and will continue to renew this training in compliance with hospital policies.

To further protect the privacy of the study participants, we will obtain a Certificate of Confidentiality from the US Department of Health and Human Services. With this certificate in place, the researchers cannot be forced to turn over identifying information about a study participant in any federal, state, or local criminal, administrative, legislative, or other proceedings. This certificate does not prevent a study participant from volunteering to turn over their research information nor does it prevent researchers from providing research-related information to others when requested by the study participant.

## Results

This is an ongoing study. The developmental interviews took place from February to April 2018. Recruitment for the RCT phase began in November 2018 and closed in November 2019. Follow-up data is still being collected. Therefore, analysis of the RCT has yet to begin.

Based on input from the qualitative interviews, content was selected to be more diverse and inclusive and less tailored to young BMSM. Participant preference for videos that more accurately reflected social interactions in the South was also considered when choosing content for the RCT. Furthermore, interview participants were asked what time of day they would want to receive the texts, so texts are sent between 11 am and 4 pm. There have been no significant changes to the videos and links that are given as intervention content since the beginning of the study. However, in the initial protocol, participants were to receive follow-up queries on days they received content asking them to rate the links. Lack of participant interest in responding to queries led the team to remove this from procedures. Currently, there are no prompts to remind participants to engage with content.

At the conclusion of recruitment, 65 participants were randomized in total (33 to the intervention and 32 to ESC). The goal was to randomize 33 participants to each arm of the study (66 in total). However, three individuals who completed baseline were withdrawn from the study prior to randomization. Demographics for the 68 participants, taken at baseline, are presented in Table 2. The average age of participants is 23.9 years. Individuals predominately identify as male; black, African American, or Haitian; and not of Hispanic or Latino descent, which reflects the eligibility criteria. Most of the sample received a high school diploma or General Educational Diploma and are either currently employed or a student. While yearly income ranges from less than $10,000 to $79,999, many participants make less than $40,000.

Of those who have been randomized, 3 were withdrawn from the study, 2 were removed from the intervention arm for seroconverting during the trial, and 1 was removed from the ESC arm for no longer self-identifying as an MSM. Until follow-up concludes, we cannot determine the effect of treatment assigned on the desired outcomes.

**Table 2:** Pre-exposure prophylaxis mobile randomized controlled trial demographics (n=68).

Variable		Value
Age in years, mean (SD)		23.9 (4.8)
**Gender identity, n (%)**		
	Male	67 (99)
	Gender nonconforming	1 (2)
**Race, n (%)**		
	American Indian or Alaskan Native	3 (4)
	Black, African American, or Haitian	62 (91)
	Multiracial	3 (4)
**Hispanic or Latino, n (%)**		
	Yes	1 (2)
	No	67 (99)
**Highest level of education, n (%)**		
	Some high school	1 (2)
	High school graduate or GED^a^	42 (62)
	College degree or higher	20 (29)
	Other	5 (7)
**Employment status, n (%)**		
	Employed full-time	28 (41)
	Employed part-time	13 (19)
	Student	13 (19)
	Disabled/unable to work	1 (2)
	Unemployed	13 (19)
**Annual income, n (%)^b^**		
	$0-$9,999	28 (47)
	$10,000-$19,999	11 (18)
	$20,000-$29,999	9 (15)
	$30,000-$39,999	7 (12)
	$40,000-$49,999	3 (5)
	$50,000-$59,999	0 (0)
	$60,000-$69,999	1 (2)
	$70,000-$79,999	1 (2)

^a^General Educational Diploma.

^b^Eight participants (12%) did not provide information on annual income.

## Discussion

### Review

The PrEP mobile messaging intervention aims to inform BMSM in the southern United States on the purpose and beneficial impacts of PrEP while reducing stigma about this method of HIV prevention. Through digital material aligned with an IMB theoretical framework, the intervention attempts to motivate participants to engage in PrEP-related care. This cost-effective, easy-to-use, interactive PrEP/HIV prevention mobile messaging intervention could provide BMSM in the southern United States with the information and motivation they need to schedule a PrEP services appointment and take preventative measures in the high-risk environment of the South. No other study has sought to improve this aspect of PrEP-related care, yet attendance at a PrEP services appointment is a crucial first step in the prevention timeline.

Moreover, the intervention is cost effective and easily adapted and tailored to participants. Free and publicly available online content will be selected through in-depth interviews. Selected online material will address information, motivation, behavioral skills, cultural barriers (such as stigma), and structural barriers (such as payment). In this way, the PrEP/HIV prevention mobile messaging intervention is made to be more effective for its target population.

Despite being developed for BMSM, the addition of diverse and inclusive material based on developmental feedback allows the intervention to be generalizable to other populations. Furthermore, the protocol’s low cost and ease of use would make the intervention easy to apply in routine settings outside of a small RCT. However, to do so would likely require a shift from the SMART platform to local agencies sending texted information by cellphone. More individuals would then need to be trained on the protocol.

### Limitations

Some limitations should be noted regarding this protocol. This trial is unblinded, which may lead participants to alter how they report on study outcomes since they are aware of their treatment group. They may also be more or less inclined to seek out additional information or be lost to follow-up. While the follow-up questionnaire asks participants how many of the links or videos they watched, this report may be inaccurate due to social desirability bias. Therefore, it will be difficult to ascertain if results are biased due to nonuse of intervention content.

### Conclusion

With the large numbers of youth possessing smartphones, the PrEP mobile messaging intervention is a promising route in the effort to increase PrEP uptake for BMSM living in the southern United States. Digital systems cross geographic and interpersonal barriers and can engage at-risk populations. The results of this study can signify the role that interactive digital interventions can play in HIV prevention and clinical care.

## Appendix

Multimedia Appendix 1 Consent to participate in research.

Multimedia Appendix 2 Peer-reviewer report from the National Institutes of Health.

## Abbreviations

BMSM: black men who have sex with men
CDC: Centers for Disease Control and Prevention
ESC: enhanced standard of care
HIPAA: Health Insurance Portability and Accountability Act
IMB: information-motivation-behavioral skills
MSM: men who have sex with men
PrEP: pre-exposure prophylaxis
REDCap: Research Electronic Data Capture
RCT: randomized controlled trial
SMART: Study Management and Retention Toolkit
STI: sexually transmitted infection
UMMC: University of Mississippi Medical Center
