# Supplementary material for: A Mobile Intervention to Improve Uptake of Pre-Exposure Prophylaxis for Southern Black Men Who Have Sex With Men: Protocol for Intervention Development and Pilot Randomized Controlled Trial
Source: JMIR Res Protoc. 2020 Feb 20;9(2):e15781. doi: 10.2196/15781 (PMC7059079; doi:10.2196/15781)
Supplement: Multimedia Appendix 1 [file resprot_v9i2e15781_app1.pdf]

CONSENT TO PARTICIPATE IN RESEARCH

The University of Mississippi Medical Center

Study Title:	A Mobile Intervention to Improve Uptake of PrEP for Southern Black MSM: Randomized Controlled Phase

Principal Investigator(s):

Mississippi –	Leandro Mena M.D., M.P.H.<br>University of Mississippi Medical Center<br>Jackson, MS
Rhode Island –	Laura Whiteley, M.D.<br>Brown University<br>Providence, RI

Site(s):	Open Arms Healthcare Center

Introduction

You are being invited to take part in this research project because you are a male age 18-35 years (assigned male at birth) who is not currently taking pre-exposure prophylactic (PrEP) medication to prevent HIV

Purpose

The purpose of this study is to evaluate if a mobile messaging intervention improves HIV-related knowledge and attitudes and attendance at PrEP appointments.

Procedures

If you agree to participate in this study, you will have three appointments over 4 months.

During your first appointment, you will complete a questionnaire regarding demographic information, sexual and drug behaviors, adherence to medical treatment, and HIV knowledge and attitudes. You may choose not to answer any questions that make you uncomfortable.

University of Mississippi Medical Center
FWA00003630
Protocol: 2017-0169
Approved: 12/03/2018
Expires: 10/21/2019

Also during your first study appointment, you will be assigned, by chance, to participate in one of the following two study groups:

(1) Group One: You will receive text with intervention content and follow-up questions over the next 4 weeks, on your cell phone. You will receive approximately 8-16 text messages with links to web content and questions about PrEP and HIV Transmission, HIV prevention, and correcting misconceptions about HIV and PrEP. Texts will be sent twice a week over the next 4 weeks.

OR

(2) Group Two: You will be given feedback regarding your PrEP eligibility, current risk behavior, and future plans. You will also be given an informational handout about PrEP, shown a brief video, and given contact information for the Clinic Care Coordinator.

All participants will have follow-up appointments at 5 weeks and 17 weeks with a member of the research team at Open Arms Healthcare Center. Study personnel will contact you to confirm your research appointments prior to the scheduled week 5 and week 17 dates. During these appointments we will ask you to complete a

questionnaire about your demographic information, sexual and drug behaviors, how you manage your medication(s), and HIV knowledge and attitudes. We will also review your medical record at Open Arms Healthcare Center and collect information related to the PrEP care that you may receive during your participation in the study.

Length of Participation

Your participation in this study will last about 4 months.

Risks

You may experience some psychological distress or emotional discomfort while answering some of the questions asked on the questionnaires regarding sexuality, PrEP, and medical care or viewing some of the mobile content about those topics. You may choose to stop completing the questionnaire at any time and do not have to answer any questions that make you feel uncomfortable.

Although every effort will be made to keep your information confidential, there is the risk of breach of confidentiality. A breach of confidentiality could occur if

information about your identity is given to a third party. To protect your identity, we will keep personal information about you private.

Benefits

You will not receive a direct benefit from being in this research study. We hope to learn information that may help others in the future. The information we gain may be used to improve the effectiveness of interventions to improve knowledge and engagement in PrEP-related care.

Alternatives

You do not have to be enrolled in this study to receive treatment or information about HIV or PrEP. If you choose not to participate in this study, information about PrEP and HIV risk prevention can be provided to you through the health educators at Open Arms Healthcare Center

Costs

There will not be any additional costs to you if you participate in this study.

Research-related injury

In the case of injury or illness resulting from your participation in this study, medical treatment is available to you at the University of Mississippi Medical Center. You will be charged the usual and customary charges for any such treatment you receive.

Compensation

You will receive:

- $50 for completing the first visit.
- $40 for completing each follow-up visit (5 and 17 weeks)
- $10 for confirming your research appointment and contact information (prior to week 5's visit), and
- $10 for confirming your research appointment and contact information (prior to week 17's visit)

Voluntary Participation

Your participation in this study is **voluntary**. If at any point you decide to discontinue participation in this study, you will not suffer a penalty or loss of benefits to which you are otherwise entitled.

Withdrawal

You may choose to stop your participation in this study at any time**.** If you decide to withdraw the information already collected about you may still be used in this study but additional information will not be collected. Your decision to stop your participation will have no effect on the quality of medical care you receive at the University of Mississippi Medical Center**.** Your participation in this study can be stopped at any time by the study doctor without your consent due to not complying with study procedures or if you become HIV positive.

New Information

You will be told of any new information we learn during your participation in this study that may affect your willingness to participate.

Confidentiality

Every effort will be made to keep the information we learn about you private. Study personnel, the study sponsor, (Lifespan, National Institute of Child Health and Human Development and the National Institutes of Health), the Food and Drug Administration (FDA), the Office for Human Research Protections (OHRP) and the University of Mississippi Medical Center's Office of Grants and Contracts, Institutional Review Board (IRB) and Office of Integrity and Compliance may review the study records.

Protected Health Information

Protected health information is any personal health information through which you can be identified. The information collected in this study includes: your name, address, ZIP code, phone number, health insurance, education level, information related to risk behaviors, medical record number, clinical history, and lab results. A decision to participate in this research means that you agree to the use of your health information for the study described in this form. This information will not be released beyond the purposes of conducting this study. The information collected for this study will be kept indefinitely. While this study is ongoing you may not have access to research information, but you may request it after the research is completed.

If the study results are published your name or other information that could identify you will _not_ be released beyond the purposes of conducting this study.

Your medical information and records, once disclosed, may be redisclosed and may no longer be protected by the Privacy Standards of the Health Insurance. Portability and Accountability Act (HIPAA), which is a federal regulation designed to protect medical information, including medical information and records created through research.

You have the right to cancel this authorization at any time by providing the

study doctor with a written request to cancel the authorization at the following address: Dr. Leandro Mena, University of Mississippi Medical Center, 2500 North State Street, Jackson, MS 39216 or you may call (601) 984-5560 (answering service will take calls after hours and on weekends). If you cancel this authorization, medical information and records about you that were created before the authorization was cancelled will still be used and disclosed as needed to preserve the integrity of the study.

If you do not sign this consent document, you will not be allowed to participate in this study.

Number of Participants

We expect 66 participants to enroll in this study.

Questions

If you have questions about this study or need to report any problems, side effects, or injuries, please call Dr. Leandro Mena at 601-984-5560 (the answering service will take calls after hours and on weekends).

You may discuss your rights as a research participant with the Chairman of the University of Mississippi Medical Center's Institutional Review Board, 2500 North State Street, Jackson, Mississippi 39216; telephone, 601 984-2815; facsimile, 601 984-2961. The Institutional Review Board is a group of people not involved with this study who have reviewed the study to protect your rights.

You will be given a copy of this consent document to keep for your records after it has been signed.

Statement of Participation

I have been told about this study and the possible risks and benefits. My participation is voluntary and I may withdraw at any time without any penalty or loss of benefits to which I am entitled, including medical care at the University of Mississippi Medical Center.

By signing this form I am not giving up any legal I may have.

____
Participant's Printed Name

____
Participant's Signature

____
Date

____
Printed Name of Person Obtaining Consent

____
Signature of Person Obtaining Consent

____
Date

I acknowledge that the participant identified above has been entered into this study, with properly obtained informed consent.

____
Signature of Investigator

____
Date

University of Mississippi Medical Center
FWA00003630
Protocol: 2017-0169
Approved: 12/03/2018
Expires: 10/21/2019
