# Supplementary material for: A Mobile Intervention to Improve Uptake of Pre-Exposure Prophylaxis for Southern Black Men Who Have Sex With Men: Protocol for Intervention Development and Pilot Randomized Controlled Trial
Source: JMIR Res Protoc. 2020 Feb 20;9(2):e15781. doi: 10.2196/15781 (PMC7059079; doi:10.2196/15781)
Supplement: Multimedia Appendix 2 [file resprot_v9i2e15781_app2.pdf]

SUMMARY STATEMENT
(Privileged Communication)

PROGRAM CONTACT:
Michael Stirratt
240-627-3875
stirrattm@mail.nih.gov

Release Date: 04/14/2016 12:15 PM
Revised Date:

Application Number: 1 R34 MH111342-01

Principal Investigators (Listed Alphabetically):

MENA, LEANDRO ANTONIO
WHITELEY, LAURA B (Contact)

Applicant Organization: RHODE ISLAND HOSPITAL

Review Group: BSPH
Behavioral and Social Science Approaches to Preventing HIV/AIDS Study Section

Meeting Date: 03/14/2016
Council: MAY 2016
Requested Start: 07/01/2016
RFA/PA: PA14-179
PCC: 9A-ASGA
Dual IC(s): AI

Project Title: A Mobile Intervention to Improve Uptake of PrEP for Southern Black MSM

SRG Action: Impact Score:40 Percentile:35 +
Next Steps: Visit http://grants.nih.gov/grants/next_steps.htm
Human Subjects: 30-Human subjects involved - Certified, no SRG concerns
Animal Subjects: 10-No live vertebrate animals involved for competing appl.
Gender: 3A-Only men, scientifically acceptable
Minority: 2A-Only minorities, scientifically acceptable
Children: 1A-Both Children and Adults, scientifically acceptable
Clinical Research - not NIH-defined Phase III Trial

Project Year	Direct Costs Requested	Estimated Total Cost
1	150,000	245,877
2	150,000	245,877
3	150,000	245,877
TOTAL	450,000	737,630

ADMINISTRATIVE BUDGET NOTE: The budget shown is the requested budget and has not been adjusted to reflect any recommendations made by reviewers. If an award is planned, the costs will be calculated by Institute grants management staff based on the recommendations outlined below in the COMMITTEE BUDGET RECOMMENDATIONS section.

1R34MH111342-01 WHITELEY, LAURA

RESUME AND SUMMARY OF DISCUSSION: The applicant seeks to develop and test the efficacy of a mobile messaging intervention to promote PrEP uptake and reduce HIV risk behaviors among Black men who have sex with men (BMSM) in Mississippi compared to standard of care. BMSM are the most in that population; moreover, the South displays among the highest HIV incidence in the US among these MSM. As such any intervention that would improve PrEP uptake and reduce HIV risk behaviors in that population would have a significant impact on public health. This intervention, while not innovative, makes use of a well-worn text messaging approach to send various HIV prevention related video links related to prevention (8 over the course of 4 weeks). Although these videos are publicly available, their relevance to the PrEP eligible MSM to whom they will be disseminated will have been identified based on in-depth interviews. The application is theoretically grounded in the IMB model; other strengths include its outstanding team of investigators, a potentially scalable intervention, supportive formative research, and the use of a platform that should ensure ready access to the intervention. Nevertheless, the application was found deficient in the following: while the IMB model is an appropriate framework for the intervention, the emphasis here is more on the informational aspect with little to no attention devoted to the motivation and behavior components. As such, the model is not well integrated with the aims of the study. Moreover, there was some disagreement over the communication approach around the web based intervention; namely, the communication is unidirectional. Once messages are sent to participants, how are their questions addressed? It is also not clear how the intervention will help BMSM address structural barriers that may play a large role in their uptake of PrEP. The application's many strengths outweigh its weaknesses; however, the latter reduced the committee's assessment of the potential impact of the research as implemented to moderate at best.

DESCRIPTION (provided by applicant): Despite advancements supporting the efficacy and availability of Pre-Exposure Prophylaxis (PrEP), uptake of PrEP has been slow for young Black men who have sex with men (BMSM) living in the Southern United States. Eight of the ten states with the highest rates of new HIV infections are located in the South. Jackson, MS (the site of this proposed project) has the second highest AIDS diagnosis rate in the nation, and the third highest rate of HIV infection for young Black men. This study will develop and test an engaging and cost effective mobile messaging intervention to improve uptake of PrEP for BMSM (18-35 yrs) living in Jackson, MS. BMSM eligible for PrEP, receiving care at the University of Mississippi Medical Center STI/HIV testing clinics, will be texted links to publicly available, interactive websites with information and motivational materials about PrEP and HIV prevention. Links will be sent to PrEP eligible BMSM by 8 text messages over 4 weeks. The material used in the intervention will be consistent with the Information Motivation Behavior (IMB) Model. Creation and adaptation of the Intervention (e.g. choosing which publicly available links to send) will occur from in-depth interviews (n = 30) with PrEP eligible BMSM receiving care in the STI/HIV testing clinics. A small randomized controlled pilot study among 66 new participants in the clinics will examine the preliminary efficacy of the intervention compared to enhanced standard of care on PrEP uptake and HIV risk behaviors. It is hypothesized that, compared to BMSM subjects receiving enhanced standard of care, BMSM receiving the PrEP Mobile Messaging Intervention will show decreased HIV risk behavior at 4 and 16 weeks, and greater PrEP uptake at 16 and 24 weeks. Black MSM seen at UMMC STI/HIV testing clinics, who are candidates for PrEP but do not start PrEP, receive 8 text messages over 4 weeks with engaging links to PrEP and HIV prevention content. Information about PrEP Motivation for PrEP uptake PrEP uptake and engagement in care HIV/STI risk behaviors

PUBLIC HEALTH RELEVANCE: This study will develop and test a mobile messaging intervention to improve uptake of a medication to prevent HIV (known as Pre-exposure Prophylaxis, PrEP) and to decrease HIV risk behaviors for Black men who have sex with men living in the Southern U.S. The mobile messaging intervention will be composed of free, publicly available links to interactive websites

and YouTube videos. The links can be easily text messaged and viewed on each participant's phone. While looking at the links, participants will gain information about PrEP and their health, improve motivation for engagement in PrEP related care, and build skills for healthy behaviors. If the intervention is found to be effective, it can be tested in a larger study and then disseminated.

CRITIQUE 1:

Significance: 2
Investigator(s): 1
Innovation: 5
Approach: 5
Environment: 2

Overall Impact: In this submission, the investigators propose developing a mobile messaging intervention for BMSM that aims to improve uptake of PrEP while decreasing risk behaviors. The investigators will use publicly available videos that are accessed through links and which can be texted. Strengths of the proposal include focus on an important population in a region with high rates of HIV. Weaknesses include limited focus on behavior change and attention to other structural factors impacting HIV acquisition in this population. Overall this is an important population and work examining a potentially significant problem; however, views of videos will likely have limited impact on increasing PrEP uptake and retention in this population.

1. Significance:

Strengths

- Focus on increasing PrEP uptake among BMSM
- Working within contexts that have high rates of HIV

Weaknesses

- None Noted

2. Investigator(s):

Strengths

- Strong group that has worked together on previous projects

Weaknesses

- None Noted

3. Innovation:

Strengths

- None Noted

Weaknesses

- Intervention design for BMSM in the South is not innovative
- IMB is not innovative

4. Approach:

Strengths

- Formative research will guide subsequent steps in the project
- Tied to a major PrEP clinic in the South.

Weaknesses

- IMB is not fully utilized – more about information, not much on motivation and behavior change. Sending links will have an impact? Not sure how links will address structural/economic changes or how they will address stigma. What if links are not consistent with what is needed?
- 16 month window is long. What if the client takes PrEP and then stops PrEP before window is up.
- Theoretically defined and tailored messages are not up to investigators since they will be drawn from existing messages.
- Information is in one direction. What options exist if client has questions about the link. Communication support is not there. How can messages be tailored to where people are at?
- M and B probably require more interaction
- Underpowered for PrEP uptake because power is based upon 3 assessments, but only two are mentioned
- Numbers not provided of how many coming to clinic and how many are eligible and engaged.

5. Environment:

Strengths

- Well-funded research institution with support for the research proposed.

Weaknesses

- None Noted

Protections for Human Subjects:

Acceptable Risks and/or Adequate Protections

Data and Safety Monitoring Plan (Applicable for Clinical Trials Only):

Acceptable

Inclusion of Women, Minorities and Children:

- Sex/Gender: Distribution justified scientifically
- Race/Ethnicity: Distribution justified scientifically
- Inclusion/Exclusion of Children under 21: Including ages < 21 justified scientifically

Vertebrate Animals:

Not Applicable (No Vertebrate Animals)

Biohazards:
Not Applicable (No Biohazards)

Budget and Period of Support:
Recommend as Requested:

CRITIQUE 2:

Significance: 3
Investigator(s): 3
Innovation: 4
Approach: 4
Environment: 1

Overall Impact: This proposal seeks to conduct formative research to develop and pilot test a mobile phone-based messaging intervention designed to increase uptake of PrEP among Black men who have sex with men (BMSM). The study will take place in Jackson, MS, which has very high rates of HIV infection among MSM. Guided by the Information, Motivation, Behavioral Skills model, the proposed study includes conducting in-depth interviews with BMSM, and implementing a pilot RCT to examine effects of the newly developed text messaging intervention in increasing PrEP uptake. The proposed intervention is relatively low cost, and is implemented via widely accessible technologies (i.e., text messages, YouTube content, etc.). This is a strong and innovative feature of the study. Likewise, implementing a PrEP uptake intervention in Jackson, MS is warranted given the HIV disparities affecting MSM in the South. While there are several strong features to the study, there are also weaknesses that reduce the overall impact of the intervention. As described, the intervention appears more an add-on intervention than a standalone one. Also, it addresses structural barriers in a very modest way (i.e., by providing information to intervention participants). Though the application is strong, as implemented, its impact is likely to be moderate.

1. Significance:

Strengths

- As noted by the investigators, some of the highest HIV infections rates are in the South. The setting where the proposed study will take place (i.e., Jackson, MS) increases the significance of the research.
- The intervention utilizes easily accessible technologies (i.e., text messages linking to YouTube videos and mobile phone accessible websites). The researchers accurately note that this could enhance dissemination of the proposed intervention given its potentially low cost.
- The investigators show that despite efforts to increase PrEP uptake among eligible BMSM in Jackson, uptake remains low. This gives rationale and significance to the proposed study.
- The intervention format (i.e., text message-based) is well suited for access by the target population. Young men who have sex with men (MSM), notably those who are minorities, have high level of access to and use of cell phone technologies.

Weaknesses

- The intervention may be best as an add-on feature to existing linkage to care/prevention interventions but not as a standalone intervention.
- The provision of information is most likely not enough to address structural barriers in accessing PrEP for BMSM in this community. The proposed intervention is unlikely to comprehensively address structural barriers to accessing primary care/PrEP.

2. Investigator(s):

Strengths

- Dr. Whiteley has experience conducting research on technology-based approaches to HIV prevention among racial/ethnic minority populations.
- Members of the investigative team have expertise in technology-based interventions, the provision of clinical care to BMSM, and qualitative research. This makes them well-prepared to conduct the formative research proposed.

Weaknesses

- Dr. Mena has little experience leading NIH-funded studies as a PI.

3. Innovation:

Strengths

- Utilization of basic text messages that link to free, easily accessible web content is a novel feature of the intervention, and also enhances the potential for dissemination.

Weaknesses

- There is limited information to support the idea that a series of text messages provided over the course of four weeks will sufficiently motivate participants to take up PrEP. Given that they are eligible and presumably have been offered PrEP, there may be reasons for lack of uptake that are not about behavior or attitudes, but perhaps tied to other structural level factors.

4. Approach:

Strengths

- The intervention content has been identified and the formative research that will be conducted to test and add to it is well thought out.
- The research team has compelling pilot data that supports the acceptability and feasibility of the proposed intervention.
- All BMSM at the Jackson clinic who are eligible for PrEP according to current treatment guidelines will be eligible for the proposed study.
- The primary and secondary outcomes, and proposed moderators to the intervention's effects, are well thought out.

Weaknesses

- Limited information is provided about how the investigators will handle change in phone numbers and/or loss of service among participants.
- Given that 40% of MSM currently show up for PrEP related appointments (and presumably take up use of PrEP), the intervention effect size may need to be particularly robust in order for the

pilot trial to be able to observe significant differences between the intervention and control groups.

- Related to the point above, the power analysis is inconsistent with current data (given that only 10% of participants in the control condition take up PrEP based on the analysis). This is a minor concern, however, given the stage of the research.

5. Environment:

Strengths

- The resources of the University of Mississippi Medical Center, Crossroads Clinic, and the Brown Center CFAR will provide a high level of resources to the two PIs as they implement the proposed intervention.
- Conducting this research study in Jackson, MS is an important feature of the study that increases its overall impact. There has been a dearth of HIV prevention and intervention research conducted in the South, and this area of the U.S. has marked disparities in HIV-related health outcomes.

Weaknesses

- None noted.

Protections for Human Subjects:

Acceptable Risks and/or Adequate Protections

- The plan for protecting human subjects is adequate.

Data and Safety Monitoring Plan (Applicable for Clinical Trials Only):

Acceptable

- A DSMP is provided.

Inclusion of Women, Minorities and Children:

- Sex/Gender: Distribution justified scientifically
- Race/Ethnicity: Distribution justified scientifically
- Inclusion/Exclusion of Children under 21: Including ages < 21 justified scientifically
- The study plans on enrolling 96 young BMSM between the ages of 18 - 35 years.

Vertebrate Animals:

Not Applicable (No Vertebrate Animals)

Biohazards:

Not Applicable (No Biohazards)

Budget and Period of Support:

Recommend as Requested:

CRITIQUE 3:

Significance: 1
Investigator(s): 3
Innovation: 4
Approach: 5
Environment: 2

Overall Impact: This R34 application will develop and test a mobile app to increase PrEP uptake among Black MSM in the South. HIV among Black MSM in the South is a serious problem and intervention that reduces it could be highly significant. There are some relatively minor problems with the approach that need to be addressed. Nonetheless, the strengths of the application outweigh its weaknesses.

1. Significance:

Strengths

- Young Black MSM represent a critical risk group for HIV in the United States
- If proven efficacious, the proposed mobile intervention using publicly available videos has the potential to be scaled-up widely

Weaknesses

- None Noted

2. Investigator(s):

Strengths

- The team of investigators seems to have appropriate experience and expertise in all of the methods and techniques they propose to use in the project.
- The multiple PI plan is justified.

Weaknesses

- The multiple PIs each have 10% per year, but the commitment of some of the co-investigators is quite low.

3. Innovation:

Strengths

- Point #3, using existing publicly available internet content for a PrEP intervention is innovative.
- Point #5, addressing stigma and structural barriers in a PrEP intervention is somewhat innovative.

Weaknesses

- Points #1, #2 & #4 listed under innovation relate to significance, not innovation.
- Point #6 (using theory) is good practice in intervention development, but it is not innovative.

4. Approach:

Strengths

- The two stage process for developing and testing the intervention is a strength.
- The use of medical records to assess PrEP use is a strength

Weaknesses

- The data collected by the technology for measuring fidelity and dosage may be difficult to interpret. It will be able to distinguish between multiple visits/views by the same participant from views by separate participants. If men view these videos on their phones they may be interrupted, cut the video short and watch it later if they find it engaging.
- The applicants suggest that the chart review at 24 weeks may increase power, however, it seems just as likely that it could reduce power if there is much diffusion of the intervention across study arms or if participants discontinue PrEP.

5. Environment:

Strengths

- The University of Mississippi Medical Center is a suitable clinical site for the application.
- The Brown University medical school, school of public health and associated hospitals provide the necessary resources for the study.

Weaknesses

- None Noted

Protections for Human Subjects:

Acceptable Risks and/or Adequate Protections

- In addition to data collection, it seems like there is a possibility that someone could have an adverse reaction to some of the videos. It may be helpful to include information in the emails that contain the links to the videos.

Data and Safety Monitoring Plan (Applicable for Clinical Trials Only):

Acceptable

- The data and safety monitoring plan is adequate.

Inclusion of Women, Minorities and Children:

- Sex/Gender: Distribution justified scientifically
- Race/Ethnicity: Distribution justified scientifically
- Inclusion/Exclusion of Children under 21: Including ages < 21 justified scientifically
- The study will include children between the ages of 18 and 21.

Vertebrate Animals:

Not Applicable (No Vertebrate Animals)

Biohazards:
Not Applicable (No Biohazards)

Resource Sharing Plans:
Acceptable

- There is no resource sharing plan, but it may not need one.

Budget and Period of Support:
Recommend as Requested:

THE FOLLOWING SECTIONS WERE PREPARED BY THE SCIENTIFIC REVIEW OFFICER TO SUMMARIZE THE OUTCOME OF DISCUSSIONS OF THE REVIEW COMMITTEE, OR REVIEWERS' WRITTEN CRITIQUES, ON THE FOLLOWING ISSUES:

PROTECTION OF HUMAN SUBJECTS: ACCEPTABLE

INCLUSION OF WOMEN PLAN (G3A): ACCEPTABLE

INCLUSION OF MINORITIES PLAN (M1A): ACCEPTABLE

INCLUSION OF CHILDREN PLAN (C1A): ACCEPTABLE

COMMITTEE BUDGET RECOMMENDATIONS: The budget was recommended as requested.

Footnotes for 1 R34 MH111342-01; PI Name: WHITELEY, LAURA B

+ Derived from the range of percentile values calculated for the study section that reviewed this application.

NIH has modified its policy regarding the receipt of resubmissions (amended applications). See Guide Notice NOT-OD-14-074 at http://grants.nih.gov/grants/guide/notice-files/NOT-OD-14-074.html. The impact/priority score is calculated after discussion of an application by averaging the overall scores (1-9) given by all voting reviewers on the committee and multiplying by 10. The criterion scores are submitted prior to the meeting by the individual reviewers assigned to an application, and are not discussed specifically at the review meeting or calculated into the overall impact score. Some applications also receive a percentile ranking. For details on the review process, see http://grants.nih.gov/grants/peer_review_process.htm#scoring.

MEETING ROSTER
Behavioral and Social Science Approaches to Preventing HIV/AIDS Study Section
AIDS and Related Research Integrated Review Group
CENTER FOR SCIENTIFIC REVIEW

BSPH
03/14/2016 - 03/15/2016

CHAIRPERSON(S)

FLYNN, PATRICK M, PHD
PROFESSOR AND DIRECTOR
INSTITUTE OF BEHAVIORAL RESEARCH
DEPARTMENT OF PSYCHOLOGY
TEXAS CHRISTIAN UNIVERSITY
FT WORTH, TX 76109

MEMBERS

ALLEN, SUSAN A, MD, MPH
PROFESSOR
DEPARTMENT OF PATHOLOGY
AND LABORATORY MEDICINE
EMORY UNIVERSITY
ATLANTA, GA 30322

BANKOLE, AKINRINOLA, PHD
DIRECTOR
INTERNATIONAL RESEARCH
THE GUTTMACHER INSTITUTE
NEW YORK, NY 10005

BLANKENSHIP, KIM M, PHD
PROFESSOR AND CHAIR
DEPARTMENT OF SOCIOLOGY
AMERICAN UNIVERSITY
WASHINGTON, DC 20016

BLUTHENTHAL, RICKY N, PHD *
PROFESSOR
DEPARTMENT OF PREVENTIVE MEDICINE
INSTITUTE FOR PREVENTION RESEARCH
KECK SCHOOL OF MEDICINE
UNIVERSITY OF SOUTHERN CALIFORNIA
LOS ANGELES, CA 90033

CHAMPION, JANE DIMMITT, PHD, DNP, FAAN *
PROFESSOR
SCHOOL OF NURSING
UNIVERSITY OF TEXAS AT AUSTIN
AUSTIN, TX 78701

CHARLEBOIS, EDWIN DUNCAN III, PHD, MPH
PROFESSOR
DEPARTMENT OF MEDICINE
SCHOOL OF MEDICINE
UNIVERSITY OF CALFORNIA, SAN FRANCISCO
SAN FRANCISCO , CA 94105

DANGERFIELD, BRIAN CHARLES, PHD *
PROFESSOR
DEPARTMENT OF MANAGEMENT
SCHOOL OF ECONOMICS, FINANCE AND MANAGEMENT
UNIVERSITY OF BRISTOL
BRISTOL BS8 1TN
UNITED KINGDOM

DICKSON-GOMEZ, JULIA B, PHD
PROFESSOR
CENTER FOR AIDS INTERVENTION RESEARCH
DEPARTMENT OF PSYCHIATRY AND BEHAVIORAL MEDICINE
MEDICAL COLLEGE OF WISCONSIN
MILWAUKEE, WI 53202

DONENBERG, GERI R, PHD *
ASSOCIATE DEAN OF RESEARCH
DEPARTMENT OF PHYCHIATRY
SCHOOL OF PUBLIC HEALTH
UNIVERSITY OF ILLINOIS AT CHICAGO
CHICAGO, IL 60608

FEASTER, DANIEL J, PHD
ASSOCIATE PROFESSOR
DIVISION OF BIOSTATISTICS
DEPARTMENT OF PUBLIC HEALTH SCIENCES
MILLER SCHOOL OF MEDICINE
UNIVERSITY OF MIAMI
MIAMI, FL 33136

FUJIMOTO, KAYO, PHD *
ASSISTANT PROFESSOR
CENTER FOR HEALTH PROMOTION
AND PREVENTION RESEARCH
SCHOOL OF PUBLIC HEALTH
UNIVERSITY OF TEXAS AT HOUSTON
HOUSTON, TX 77030

HAHM, HYEOUK CHRIS, PHD *
ASSOCIATE PROFESSOR
SCHOOL OF SOCIAL WORK
BOSTON UNIVERSITY
BOSTON, MA 02215

HAMMETT, THEODORE M, PHD *
VICE PRESIDENT AND PRINCIPAL ASSOCIATE
INTERNATIONAL HEALTH DIVISION
ABT ASSOCIATES INCORPORATED
CAMBRIDGE, MA 02138

HAVENS, JENNIFER R, PHD, MPH
ASSOCIATE PROFESSOR
DEPARTMENT OF BEHAVIORAL SCIENCE
COLLEGE OF MEDICINE
UNIVERSITY OF KENTUCKY
LEXINGTON, KY 40504

HIGHTOW-WEIDMAN, LISA B, MD *
ASSOCIATE PROFESSOR
DIVISION OF INFECTIOUS DISEASES
DEPARTMENT OF MEDICINE
SCHOOL OF MEDICINE
UNIVERSITY OF NORTH CAROLINA
CHAPEL HILL, NC 27599-7030

KURTZ, STEVEN P, PHD
PROFESSOR AND DIRECTOR
CENTER FOR APPLIED RESEARCH ON SUBSTANCE USE AND HEALTH DISPARITIES
DEPARTMENT OF JUSTICE AND HUMAN SERVICES
NOVA SOUTHEASTERN UNIVERSITY
CORAL GABLES, FL 33134

MACQUEEN, KATHLEEN M, PHD
SENIOR SCIENTIST
SOCIAL AND BEHAVIORAL HEALTH SCIENCES
FHI 360
DURHAM, NC 27701

MARTINEZ-DONATE, ANA P, PHD
ASSOCIATE PROFESSOR
DEPARTMENT OF POPULATION HEALTH SCIENCES
SCHOOL OF MEDICINE AND PUBLIC HEALTH
DREXEL UNIVERSITY
PHILADELPHIA, PA 19104

NASH, DENIS, PHD
PROFESSOR
EPIDEMIOLOGY AND BIOSTATISTICS PROGRAM
SCHOOL OF PUBLIC HEALTH
CITY UNIVERSITY OF NEW YORK
NEW YORK, NY 10035

O'DONNELL, LYDIA N, EDD *
DIRECTOR
HEALTH AND HUMAN DEVELOPMENT DIVISION
EDUCATION DEVELOPMENT CENTER INCORPORATED
NEWTON, MA 02458

ROSSER, B R SIMON, PHD, MPH
PROFESSOR AND DIRECTOR
DIVISION OF EPIDEMIOLOGY AND COMMUNITY HEALTH
UNIVERSITY OF MINNESOTA
MINNEAPOLIS, MN 55454

SCHNEIDER, JOHN, MD
ASSOCIATE PROFESSOR
DEPARTMENT OF PUBLIC HEALTH SCIENCES
UNIVERSITY OF CHICAGO
CHICAGO, IL 60637

WENZEL, SUZANNE L, PHD
PROFESSOR
SCHOOL OF SOCIAL WORK
UNIVERSITY OF SOUTHERN CALIFORNIA
LOS ANGELES, CA 90089

WILSON, PATRICK ALAN-DAVID, PHD
ASSOCIATE PROFESSOR
DEPARTMENT OF SOCIOMEDICAL SCIENCES
MAILMAN SCHOOL OF PUBLIC HEALTH
COLUMBIA UNIVERSITY
NEW YORK , NY 10032-3702

ZULE, WILLIAM A, DRPH *
SENIOR HEALTH ANALYST
DEPARTMENT OF HEALTH, SOCIAL ECONOMIC RESEARCH
RESEARCH TRIANGLE INSTITUTE INTERNATIONAL
RESEARCH TRIANGLE PARK, NC 27709

MAIL REVIEWER(S)

BAUER, LANCE O, PHD
PROFESSOR
DEPARTMENT OF PSYCHIATRY
UNIVERSITY OF CONNECTICUT HEALTH CENTER
FARMINGTON, CT 06030

SCIENTIFIC REVIEW OFFICER

GUERRIER, JOSE H, PHD
SCIENTIFIC REVIEW OFFICER
CENTER FOR SCIENTIFIC REVIEW
NATIONAL INSTITUTES OF HEALTH
BETHESDA, MD 20892

EXTRAMURAL SUPPORT ASSISTANT

STROTHERS, DIARA
EXTRAMURAL SUPPORT ASSISTANT
CENTER FOR SCIENTIFIC REVIEW
NATIONAL INSTITUTES OF HEALTH
BETHESDA, MD 20892

* Temporary Member. For grant applications, temporary members may participate in the entire meeting or may review only selected applications as needed.

Consultants are required to absent themselves from the room during the review of any application if their presence would constitute or appear to constitute a conflict of interest.
